# Advances in Intercellular Communication Mediated by Exosomal ncRNAs in Cardiovascular Disease

**DOI:** 10.3390/ijms242216197

**Published:** 2023-11-11

**Authors:** Xiaoyan Zhang, Shengjie Sun, Gang Ren, Wujun Liu, Hong Chen

**Affiliations:** 1 College of Animal Science, Xinjiang Agricultural University, Urumqi 830052, China; zhangxiaoyan1106@yeah.net; 2 Key Laboratory of Animal Genetics, Breeding and Reproduction of Shaanxi Province, College of Animal Science and Technology, Northwest A&F University, Xianyang 712100, China; ssjsci@126.com (S.S.); rengang666@nwafu.edu.cn (G.R.)

**Keywords:** cardiovascular disease, exosomes, miRNAs, lncRNAs, circRNAs, intercellular communication

## Abstract

Cardiovascular diseases are a leading cause of worldwide mortality, and exosomes have recently gained attention as key mediators of intercellular communication in these diseases. Exosomes are double-layered lipid vesicles that can carry biomolecules such as miRNAs, lncRNAs, and circRNAs, and the content of exosomes is dependent on the cell they originated from. They can be involved in the pathophysiological processes of cardiovascular diseases and hold potential as diagnostic and monitoring tools. Exosomes mediate intercellular communication, stimulate or inhibit the activity of target cells, and affect myocardial hypertrophy, injury and infarction, ventricular remodeling, angiogenesis, and atherosclerosis. Exosomes can be released from various types of cells, including endothelial cells, smooth muscle cells, cardiomyocytes, fibroblasts, platelets, adipocytes, immune cells, and stem cells. In this review, we highlight the communication between different cell-derived exosomes and cardiovascular cells, with a focus on the roles of RNAs. This provides new insights for further exploring targeted therapies in the clinical management of cardiovascular diseases.

## 1. Introduction

Cardiovascular disease (CVD) includes diseases of the heart muscle, blood vessels, or interstitial tissues. The prevalence of CVD has risen dramatically over the past two decades, making it one of the major issues of global concern and imposing an enormous social and economic burden. In 2012, approximately 17.5 million people died from CVD, and this number is expected to increase to 23 million by 2030. As the heterogeneity and complexity observed in CVD progression increases, the need for specific and accurate diagnosis of the disease state becomes more urgent. In this context, it is therefore of great interest to identify new targets to evaluate pathological responses or new diagnostic therapies for the early detection of people at risk of CVD. The number and characteristics of exosomes change according to the pathophysiological state of the disease and, therefore, can serve to some extent as biomarkers for diagnosis and monitoring of the disease. The identification of biomarkers with high sensitivity and specificity is necessary in the diagnosis and treatment of CVD. The study of exosomes has become a frontier and hotspot in the diagnosis and treatment of CVD.

Exosomes, as a new molecular platform for chemical signaling, have played a great role in recent years in a variety of diseases including cardiovascular diseases and tumors [1,2,3]. Exosomes are vesicle-like substances secreted by living cells with a double-membrane structure, and their diameters generally range from 30 nm to 150 nm. All cells, including those of eukaryotic and prokaryotic cells, are capable of releasing exosomes both under physiological and pathological conditions [4]. Exosomes have a similar topology to cells and contain substances such as DNA, RNA, proteins, lipids, small molecule metabolites, and cell surface proteins [5]. The reasons for cells producing exosomes and the physiological functions of exosomes are still not particularly well understood, and more research is needed to prove this. Previously, it was believed that the main function of exosomes was to remove excess or nonessential cellular components from the cell to maintain cellular homeostasis. In recent years, studies have shown that cells can selectively accumulate cellular components in exosomes through targeted and mechanism-specific-driven processes, which suggests an important role for exosomes in regulating intercellular communication [6]. In 2007, it was first discovered that exosomes could transport miRNA between cells, and this finding revealed an important transport mechanism for the functional role of ncRNAs [7]. Several studies have confirmed that exosomes are important tools for the extracellular transport of miRNA and lncRNA [8,9,10,11]. The ncRNAs carried by exosomes are highly stable in body fluids and have the potential to serve as biomarkers. There is increasing evidence that ncRNAs in exosomes are not only diverse and abundant but also highly associated with a variety of diseases such as cardiovascular diseases, central nervous system diseases, and malignant tumors [12,13,14,15,16,17]. In view of this, exosomal ncRNAs have a broad prospect in the field of disease diagnosis.

The exosomes are important bioinformatic carriers that facilitate intercellular communication and participate in the pathophysiological processes of various cardiovascular diseases. Exosomes are released by different types of cells in response to various biological and chemical stimuli, including oxidative stress [18], low pH [19], and hypoxia [20]. More importantly, these findings have introduced a new approach in cardiology, where exosomes are recognized as a new mechanism of communication between the heart and other organs. A study has explored that the exosomes involved in atherosclerosis originated from various cell types, with the majority being derived from leukocytes. Specifically, macrophages account for 29 ± 5%, lymphocytes for 15 ± 3%, granulocytes for 8 ± 1%, red blood cells for 27 ± 4%, smooth muscle cells for 13 ± 4%, and endothelial cells for 8 ± 2% [21].

## 2. Biology of Exosomes

### 2.1. The Biogenesis and Uptake of Exosomes

**Figure 1:**
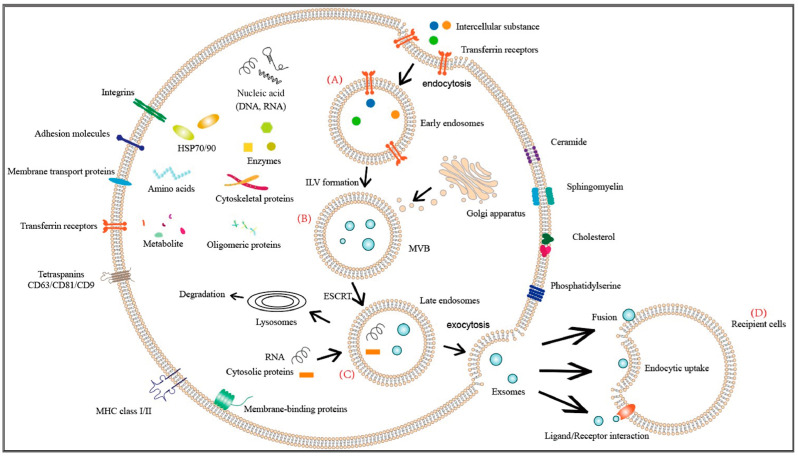
Structure, composition, secretion, and cellular entry of exosomes. Note: (A) means early endosomes, (B) means multivesicular bodies (MVBs), (C) means late endosomes and (D) means recipient cells.

The origin, synthesis, and secretion of exosomes involve the following process: the cell membrane of the parent cell forms early endosomes through endocytosis or “inward budding” (Figure 1). These early endosomes gradually mature into late endosomes and multivesicular bodies (MVBs) within the cell (Figure 1). The precursor of extracellular vesicles exists as intraluminal vesicles (ILVs) inside MVBs (Figure 1); subsequently, MVBs fuse with the cell membrane and excrete ILVs into the extracellular space through exocytosis, forming exosomes (Figure 1) [22]. As a result, their composition and structure are more complex, and they may even contain components engulfed from the extracellular matrix or culture medium. Exosomes contain proteins such as membrane transport proteins (GTPases, Annexins, Flotillin), heat shock proteins (HSP90-70), transmembrane proteins (CD81, CD63, CD9), and proteins associated with MVB biogenesis (ALIX and TSG101). They also have higher levels of membrane cholesterol and diacylglycerol [23]. The biogenesis of exosomes is regulated by two mechanisms. One is the endosomal sorting complex required for transport (ESCRT)-dependent pathway and the other is the ESCRT-independent pathway. The former involves more than 30 proteins organized into four families (ESCRT-0, I, II, III), while the latter involves proteins such as neutral sphingomyelinase 2 (nSMase2) and CD63.

### 2.2. Mechanisms of Exosome Internalization

Compared to the relatively clear “linear process” of exosome generation, the fate of exosomes is significantly complex and uncertain. If exosomes are recognized and “captured” by neighboring tissue cells, they will be taken up and utilized by the neighboring cells. If the adjacent cells do not recognize and capture the exosomes, the exosomes are transported through the circulatory system to more distant cells or tissues. The uptake and utilization of exosomes by both adjacent and distant tissue cells are mainly through several different mechanisms of action [24,25].

(1) The ligand on the membrane surface of exosomes binds to the receptor on the membrane of the recipient cells, activating the receptor-mediated signaling pathway, and the activated recipient cells ingest the contents into the cell through endocytosis. For example, exosomes derived from dendritic cells containing MHC–peptide complexes can activate T cells through homologous T-cell receptors [26]; (2) Recipient cells directly internalize exosomes through endocytosis, allowing the contents of the exosomes to be released into the cells. Some components of exosomes participate in new multivesicular body biosynthesis processes; (3) Direct fusion of the exosome membrane with the cell membrane releases the exosome contents into the cytoplasm. Since exosomes carry a number of lipid and protein components, the interaction of these exosomal membrane proteins with receptors present in the membrane of the recipient cells may trigger responses leading to cellular changes. Thus, through these processes, exosomes can interact with recipient cells to trigger the release of cargo or the induction of signaling cascades that ultimately lead to changes in cellular activity or function [27,28].

Exosomes are also primarily characterized by their ability to enter the recipient cells and directly transfer active substances protected by the lipid bilayer membrane to the target cells. The mechanisms by which exosomes are internalized by the recipient cells are numerous and controversial. Currently suggested mechanisms include cell-specific uptake, non-specific protein interactions, endocytosis, and membrane fusion [29]. Most studies suggest that the uptake of exosomes is non-specific. On the one hand, tumor-cell-derived exosomes can be taken up by multiple cells [25], and on the other hand, exosome-mediated material delivery can cross species. For example, exosomes can transfer functional mRNAs and miRNAs from mouse to human mast cells and mouse proteins can be detected in human mast cells [7]. 

The fate of exosomes is highly uncertain and influenced by various factors. There is both directed migration towards specific destinations, such as receptor-mediated specific binding, as well as aimless or random wandering. They can be engulfed non-specifically by the circulatory system and reticuloendothelial system, or their destinations can be influenced by the local microenvironment and cellular status of recipient cells. Furthermore, studies have shown that exosomes can interact with specific target cells based on their cargo and origin, efficiently releasing their contents to induce phenotypic changes in target cells [30]. Due to their ability to effectively deliver bioactive substances or artificially loaded molecular cargos, exosomes have earned a reputation as ”natural carriers”. Therefore, in-depth exploration of the generation process, secretion conditions, and biological functions is crucial in understanding their impact on the physiological and pathological processes of recipient cells, as well as their clinical applications. 

Nowadays, the secretion of exosomes has expanded to various cell types, and their significance in intercellular communication in both normal and pathological states has been well documented. Moreover, different cell types regulate exosome biogenesis according to their physiological state and release exosomes with specific lipid, protein, and nucleic-acid compositions.

## 3. ncRNAs and Cardiovascular Diseases

With the continuous development and advancement of molecular biology technology, the role of genes and RNA in cardiovascular diseases has been increasingly emphasized. Some studies have shown that ncRNAs are abundantly expressed in the cardiovascular system, and changes in ncRNA expression levels have been found in the occurrence and development of many cardiovascular diseases. ncRNAs include transfer ribonucleic acid (tRNAs), ribosome ribonucleic acid (rRNAs), long non-coding ribonucleic acid (lncRNAs), circular ribonucleic acid (circRNAs), small nuclear ribonucleic acid (snRNAs) and micro-ribonucleic acid (miRNAs), as well as other RNAs with unknown functions. It is now well established that lncRNAs, circRNAs, and miRNAs are closely associated with the development of CVDs and have been recognized as new biomarkers and potential therapeutic targets for a variety of diseases, including CVDs.

Recent studies have proposed approximately 50 miRNAs associated with essential hypertension and more than 30 miRNAs associated with heart failure and myocardial infarction, many of which could serve as promising biomarkers. miR-21 was expressed in many cell types associated with the cardiovascular system, including vascular smooth muscle cells, vascular endothelial cells, cardiomyocytes, cardiac fibroblasts, and blood [31]. miR-21 expression was closely associated with the occurrence and development of hypertension [32]. In addition, studies of cardiac dysfunction after myocardial infarction have shown that miR-21 can be involved in the occurrence of hypertension and myocardial fibrosis through the TGF-β/smad7 signaling pathway [32]. miR-19a/19b expression was up-regulated in patients with heart failure after myocardial infarction, and miR-19a/19b increased cardiac proliferation and regeneration, suggesting that there might be a compensatory mechanism for the stress response [33].

Similar to miRNAs, a large number of lncRNAs are involved in the critical regulation of a variety of cardiac diseases, highlighting their role in the occurrence and development of cardiovascular disease. It was shown that lncRNA H19 promoted the development of atherosclerosis by promoting the MAPK and NF-kB signaling pathways [34]. lncRNA Chaer was significantly down-regulated in hypoxia-treated cardiomyocytes and in the hearts of myocardial infarction [35]. In vitro overexpression of lncRNA Chaer reduced hypoxia-induced cardiomyocyte apoptosis; conversely, silencing of lncRNA Chaer promoted cardiomyocyte apoptosis [35]. In vivo overexpression of lncRNA Chaer slowed myocyte apoptosis, reduced the infarcted area, and improved cardiac function in infarcted mice, suggesting that lncRNA Chaer has a protective effect against myocardial infarction [35]. lncRNA Nron expression was elevated in the peripheral blood of myocardial infarcted patients and in hypoxia-stimulated H9c2 cells [36]. Knockdown of lncRNA Nron promoted cell viability and inhibited apoptosis in hypoxia-stimulated H9c2 cells; meanwhile, knockdown of lncRNA Nron significantly attenuated cardiac injury and improved cardiac function in myocardial infarction mice [36].

The role of circRNA in various cardiovascular diseases has also been reported. Exogenous expression of circANRIL in a rat model of coronary atherosclerosis exerted beneficial effects by lowering the levels of total cholesterol, triglycerides, ox-LDL, and pro-inflammatory and pro-apoptotic markers’ expression in endothelial cells, thereby playing a beneficial role [37]. circRNA circ_0003204 inhibited the progression of atherosclerosis through the miR-370-3p/TGFβR2/phosph-SMAD3 axis [38]. circSNRK was significantly down-regulated in myocardial infarction rats, and overexpression of circSNRK in cardiomyocytes inhibited apoptosis and promoted cell proliferation. Overexpression of circSNRK in the heart after myocardial infarction reduced cardiomyocyte apoptosis, promoted cardiomyocyte proliferation, enhanced angiogenesis, and improved cardiac function. Overall, circSNRK promotes cardiac survival and functional recovery after myocardial infarction [39].

## 4. Exosome-Mediated Intercellular Communication

Since intercellular communication is critical for maintaining tissue homeostasis and preventing disease, understanding exosome-mediated intercellular communication may provide important insights into the development of vascular pathogenesis. Over short distances, direct cell-to-cell contact can generate cellular crosstalk, whereas long-distance communication can be mediated by cytokines or hormones. Recently, a novel communication pathway mediated by exosomes has emerged as a newly discovered means of intercellular communication. Transfer of exosome contents to receptor cells has been described in a variety of cells and these exosomes act as regulators of intercellular communication between neighboring and distal cells [40]. Furthermore, the microenvironmental stimuli can influence the quantity and types of exosome contents through induction of molecular enrichment or depletion [41]. Therefore, their potential use as diagnostic, prognostic, and therapeutic markers in physiological and pathological processes has garnered immense interest [42,43,44,45]. The role of exosomes as key regulators in intravascular homeostasis and cardiovascular disease progression has been emphasized by recent studies [46,47,48,49,50,51].

### 4.1. The Role of Exosomal ncRNA from Vascular Smooth Muscle Cells in Cardiovascular Disease

The composition of the human medium and large arterial vessel wall includes a variety of cells such as endothelial cells (ECs), immune macrophages, and vascular smooth muscle cells (SMCs). The structural and functional integrity of these cells is an important guarantee of the maintenance of the normal physiological function of blood vessels. Among the various cells, ECs and SMCs especially, perform very complicated intercellular communication. They are the main components of the vascular wall and exhibit a high degree of plasticity. The communication between ECs and VSMCs can be carried out in an indirect (biochemical) manner [52]. Exosomes originating from VSMCs can inhibit vascular regeneration through an intermediary mediation of miR-16 to down-regulate vascular endothelial growth factor levels and inhibit vascular regeneration in breast cancer cells [53]. Research has explored the role of exosome-mediated transfer of miR-155 from VSMCs to ECs in inducing endothelial injury and promoting atherosclerosis [54]. The study reveals that VSMCs release exosomes carrying miR-155, which can be taken up by neighboring ECs. This transfer leads to endothelial injury characterized by compromised barrier function, increased permeability, and enhanced endothelial cell apoptosis [54].

### 4.2. The Role of Exosomal ncRNA from Endothelial Cells in Cardiovascular Disease

Abnormal proliferation of VSMCs plays an important role in the development of diabetic vascular complications. Under high glucose (HG) conditions, ECs act as the first barrier to injury stimuli, triggering multiple responses, including ECs and VSMCs crosstalk. The results showed that exosomes secreted by mouse aortic endothelial cells (MAEC) promoted HG-induced proliferation and inhibited apoptosis of VSMCs. MAEC exosomes exposed to HG could transfer circHIPK3 enriched in MAEC exosomes to VSMCs [55]. Exosomal circHIPK3 promoted VSMC proliferation and inhibited VSMC apoptosis. circHIPK3 was sponge-wiped with miR-106a-5p to deregulate its inhibition of Foxo1 expression. The increased expression of Foxo1 served as a transcription factor to promote the expression of Vcam1, which promoted the uptake of MAEC-derived exosomes by VSMCs [55].

In the presence of atherosclerosis, the amount of microRNA-92a-3p in EC exosomes was significantly increased [56]. MicroRNA-92a-3p encapsulated by EC exosomes could translocate to recipient cells and regulate target genes through a thbs1-dependent mechanism [56]. Oxidized low-density lipoprotein-treated VSMCs released exosomes encapsulating miR-505 [57]. These exosomes are ingested and internalized into macrophages, targeting and inhibiting SIRT3 in neutrophils, thereby inducing elevated levels of reactive oxygen species in neutrophils, triggering an inflammatory response, and exacerbating atherosclerosis development [57]. It was found that exosomal miRNAs mediated signaling between cardiomyocytes and ECs [58]. Prolactin hydrolyzed fragments could mediate signaling between cardiomyocytes and ECs by stimulating ECs to release exosomes containing miR-146a [58]. Acquisition of these exosomes by cardiomyocytes resulted in a decrease in metabolic activity [58]. Specifically, the researchers discovered that miRNA-143/145 played a key role in the development of atherosclerosis. miRNA-143/145 was transferred from ECs to SMCs via exosomes, mediating intercellular communication. This communication could inhibit the proliferation and migration of SMCs and maintain the integrity of the vascular endothelium, thus exerting a protective effect on the process of atherosclerosis [59].

The increased expression of lncRNA-LINC00174 in exosomes derived from mouse primary aortic ECs directly interacted with SRSF1 to suppress the expression of p53, thereby inhibiting p53-mediated autophagy and cell apoptosis, reducing ischemia-reperfusion-induced myocardial injury [59]. Knockdown of circNPHP4 inhibited heterotypic adhesion between monocytes and coronary artery endothelial cells and reduced the expression of ICAM-1 and VCAM-1. Studies on the potential mechanisms showed that circNPHP4 affected the expression of miR-1231 and its target gene EGFR. Overexpression of miR-1231 abrogated the inhibitory effect of circNPHP4 on heterotypic adhesion [60]. The article comprehensively elucidated the abnormal communication between ECs and macrophages mediated by METTL3 through the regulation of miR-93 in exosomes in smoking-induced emphysema [61]. Smoking induced the accumulation of METTL3, which in turn promoted the maturation of miR-93 in exosomes. By targeting the negative regulation of DUSP2, the JNK/MMPs pathway was activated, leading to the degradation of elastin and the development of emphysema [61].

### 4.3. The Role of Exosomal ncRNA from Cardiac Fibroblasts in Cardiovascular Disease

Cardiac fibroblasts (CFs) play an important role in the regulation of cardiac physiology during injury [62]. Emerging evidence suggests that exosomes released by CFs are one of the major components leading to cardiomyocyte hypertrophy [63,64,65,66]. Recently, several published papers have confirmed the direct involvement of fibroblasts in ec-mediated angiogenesis [67,68]. The study investigated the role of CF-derived exosomes microRNA in cardiomyocyte hypertrophy. Evaluation of miRNA content in exosomes derived from CFs revealed a relatively high abundance of miRNAs. Among them, miR-21_3p (miR-21*) could be taken up by cardiomyocytes to silence SORBS2 or PDLIM5, leading to the induction of cardiomyocyte hypertrophy [69]. In addition, CF-exo miR-23a-3p could be taken up by cardiomyocytes, resulting in the inhibition of SLC7A11 expression and the promotion of ferroptosis, ultimately contributing to the development of atrial fibrillation [70].

The role of myofibroblast-derived exosomes in inducing cardiac EC dysfunction was explored [71]. It was shown that miR-200a-3q secreted by TGF-β-activated fibroblasts targeted the VEGF-A/PIGF signaling pathway, leading to a decrease in vascular formation ability, impaired migration capability, and increased vascular permeability in cardiac ECs [71]. A research article on miRNA-423-3p derived from CF exosomes regulating cardioprotective effects after ischemia is reported [72]. The study discovered that miRNA-423-3p in CF-derived exosomes was able to attenuate cardiac damage induced by ischemia-reperfusion injury and improve cardiac function by translocating into cardiomyocytes and targeting the downstream effector RAP2C [72]. The study aimed to investigate the role of circular RNAs (circRNAs) derived from M2 macrophages (M2Ms) exosomes in the development of myocardial fibrosis. The results showed that the circRNAbe3a derived from M2Ms exosomes promoted the proliferation, migration, and phenotypic transformation of CFs by directly targeting the miR-138-5p/RhoC axis, which might exacerbate myocardial fibrosis after acute myocardial infarction [73].

### 4.4. The Role of Exosomal ncRNA from Cardiomyocyte in Cardiovascular Disease

Myocardial infarction (MI) is the leading cause of congestive heart failure and death. Hypoxia is an important trigger factor for myocardial remodeling during the development of heart disease. The mechanism by which exosomes derived from cardiomyocytes promote cardiac fibrosis through interactions between cardiomyocytes and fibroblasts was investigated. The study found that lncRNA AK139128 produced by cardiomyocytes under hypoxic conditions could be delivered to CFs through exosomes, regulating their apoptosis and proliferation processes [74]. Cardiomyocytes secreted exosomes containing miR-208a into fibroblasts, which promoted fibroblast proliferation and differentiation toward myofibroblasts by targeting Dyrk2 [75]. The contribution of miR-210-3p from myocyte-derived exosomes to atrial fibrosis in patients with atrial fibrillation was covered [76]. The findings indicated that miR-210-3p inhibited GPD1L in atrial fibroblasts, thereby regulating the PI3K/AKT signaling pathway and promoting cell proliferation and collagen synthesis [76]. By inhibiting miR-210-3p in these exosomes, the extent of atrial fibrosis could be attenuated [76]. Meanwhile, the study found that the exosome released by cardiomyocytes contained miRNA-92a, and that the exosome was able to deliver miR-92a to postischemic myofibroblasts, alleviating smad7-mediated repression of αSMA transcription and triggering phenotypic transformation towards myofibroblasts [77]. The study investigated the mechanism by which cardiomyocytes mediated anti-angiogenesis in type-2 diabetic rats by releasing exosomes for delivery to endothelial cells [78]. The results showed that cardiomyocytes transferred exosomes miR-320 to ECs, leading to the down-regulation of target genes (*IGF-1*, *Hsp20*, and *Ets2*), thereby inhibiting ECs proliferation, migration, and angiogenesis [78]. 

In 2019, the specific expression pattern of circular RNAs (circRNAs) in exosomes from cardiac tissues during ischemia/reperfusion (I/R) injury was first revealed, providing important evidence for the role of circRNAs and exosomes in cardiac I/R pathology [79]. It was found that exosomes released from hypoxia preconditioned cardiomyocytes were enriched in circHIPK3, which was delivered to cardiac microvascular ECs and inhibited *IGF-1* expression by binding to miR-29a to reduce oxidative stress-induced injury and protected cardiac microvascular ECs [80]. On the other hand, the circHIPK3 in these exosomes could increase the expression of VEGFA by inhibiting the activity of miR-29a [81]. This, in turn, accelerated cell cycle progression and proliferation of cardiac ECs, ultimately facilitating angiogenesis [81].

lncRNA-AK139128, up-regulated in hypoxia preconditioned cardiomyocyte-derived exosomes, promoted cardiac fibroblast apoptosis, and inhibited their proliferation and migration [74]. lncRNA-MALAT1 was up-regulated in exosomes derived from cardiomyocytes treated with hyperbaric oxygen, inhibiting the expression of miR-92a and relieving miR-92a-mediated inhibition of *KLF2* and *CD31* expression after myocardial infarction, thus, enhancing neovascularization and significantly reducing the infarct size [82]. Furthermore, exosomes enriched with lncRNA-ZFAS1 from cardiomyocytes have been shown to worsen cardiac fibrosis in a mouse model of chronic kidney disease through the activation of the Wnt4/β-catenin signaling pathway [83].

### 4.5. The Role of Exosomal ncRNA from Stem Cells in Cardiovascular Disease

Embryonic stem cells (ESCs) hold great promise for cardiac regeneration and have the ability to produce exosomes, and the miR-290-295 cluster, especially miR-294, is significantly enriched in ESCs exosomes [84]. The delivery of miR-294 to the heart mediated the activation of endogenous repair mechanisms, enhancing cardiac function [84]. A study evaluated the protective effects of mesenchymal stem cell (MSC)-derived exosomes on cardiomyocytes in an animal model of doxorubicin-induced cardiomyopathy [85]. This protection was achieved through the modulation of the miR-199a-3p-Akt-Sp1/p53 signaling pathway [85]. Exosomes derived from MSCs have been widely reported to have a protective effect against myocardial infarction. It was discovered that blocking exosomal miRNA-153-3p derived from bone marrow mesenchymal stem cells could activate the VEGF/VEGFR2/PI3K/Akt/eNOS pathway, thereby alleviating hypoxia-induced myocardial and microvascular damage and improving myocardial function [86]. A study has identified that exosomes derived from iPS carried various cardioprotective miRNAs, including miRNA-21 and miRNA-210 [87]. Delivery of these miRNAs modulated multiple target genes, inhibited cardiomyocyte apoptosis, and promoted cell survival and improvement of myocardial function [87]. It provides a novel therapeutic strategy of utilizing exosomes to deliver miRNAs for cardioprotection and improving prognosis in ischemic heart disease [87].

LncRNA-UCA1 expression was found to be up-regulated in hypoxia-treated MSC exosomes. In vitro experiments demonstrated that exosomal lncRNA-UCA1 mediated the protective effect on hypoxic cardiomyocytes through the miR-873-5p/interlocked apoptosis inhibitory protein (XIAP) axis [88]. MSC-derived exosomal lncRNA KLF3-AS1 ameliorated the focal death of infarcted cardiomyocytes, inhibited the release of pro-inflammatory cytokines, and ultimately reduced the area of cardiac infarction by competitively inhibiting the down-regulation of silencing information regulatory factor 1 (SIRT1) by miR-138-5p [89]. Increased lncRNA H19 in MSC-derived exosomes pretreated with atorvastatin up-regulated the expression of *VEGF* and *ICAM-1* and attenuated the apoptosis of infarcted myocardium by acting on miR-675 [90]. Meanwhile, it down-regulated the levels of *IL-6* and *TNF-α*, inhibited the excessive inflammatory response, and ultimately improved the cardiac function of infarcted hearts [90]. The expression of lncRNA-NEAT1 in human adipose MSC-derived exosomes pretreated with macrophage migration inhibitory factor (MIF) was up-regulated, which protected cardiomyocytes from H_2_O_2_-induced apoptosis by competitively inhibiting miR-142-3p and restored the expression of FOXO1 [91]. Additionally, lncRNA-UCA1 in exosomes derived from human umbilical cord mesenchymal stem cells up-regulated the anti-apoptotic factor Bcl-2 through competitive binding with miR-143, inhibiting excessive apoptosis and autophagy in the hearts of rats subjected to ischemia-reperfusion injury [92].

The study investigated whether exosomes derived from umbilical cord mesenchymal stem cells (UMSCs) can repair the heart after myocardial infarction (MI) by delivering circRNAs. The results showed that circRNA-0001273 significantly inhibited apoptosis of myocardial cells under ischemic conditions and promoted myocardial infarction repair, providing a promising reference for clinical treatment [93]. The exosomes derived from circRNA_0002113-deficient MSCs could regulate the RUNX1 nuclear relocation by sponge miR-188-3p to inhibit myocardial infarction [94]. The expression of circHIPK3 in ischemic muscles was reduced, while treatment with exosomes derived from umbilical cord mesenchymal stem cells (UMSC-Exo) significantly increased circHIPK3 expression and improved blood perfusion [95]. Functional studies demonstrated that miR-421/FOXO3a is a direct target of circHIPK3 [95].

### 4.6. The Role of Exosomal ncRNA from Adipocyte in Cardiovascular Disease

Adipocytes have been shown as a major source of exosomes containing miRNAs [96,97,98]. The results showed that exosomes released from visceral adipocytes carried high levels of miR-27b-3p, and these exosomes could activate ECs by translocating miR-27b-3p and promote endothelial inflammation and atherosclerosis by activating the NF-κB pathway through down-regulation of PPARα [99]. Exosomes released from epididymal white adipose tissue were enriched in miR-23a-3p [100]. These exosomes promoted myocardial fibrosis by translocating miR-23a-3p to fibroblasts, which were converted to myofibroblasts [100]. Meanwhile, it has been suggested that adipocyte-derived exosomes deliver lncRNA-SNHG9 to ECs, which in turn suppresses the expression of the TNF receptor-associated death domain (TRADD), thereby reducing endothelial inflammation and apoptosis [101]. This provides a potential molecular mechanism for the maintenance of endothelial function [101]. Research established that microRNA-31 presented in exosomes derived from adipose-derived stem cells could be penetrated to cardiomyocytes and inhibited *FIH1* expression, thus encouraging the activation of HIF-1α with enhancing cell survival ability, promotion of angiogenesis, and inflammatory reaction reduction, consequently diminishing the impact of myocardial infarction [102]. The exosomes released from highly obese adipocytes were enriched in miR-802-5p, which inhibited the expression of *HSP60*, altered the pathways associated with insulin signaling in cardiac myocytes, and led to the development of insulin resistance [103]. This finding not only deepens the understanding of the mechanism of insulin resistance but also provides new clues for further exploration of the treatment of obesity-related heart diseases [103].

### 4.7. The Role of Exosomal ncRNA from Immune Cells in Cardiovascular Disease

Expansion and activation of CD4+ T cells in the heart have been identified as contributors to pathological cardiac remodeling. The researchers found that the miR-142-3p in exosomes released by activated CD4+ T cells inhibited the expression of *APC*, the negative regulator of the WNT signaling pathway, promoting post-ischemic ventricular remodeling [104]. Researchers have found that the crosstalk between cardiomyocytes and macrophages is mediated by the expression of miR-34a-5p/PNUTS signaling pathway in exosomes to exert pro-aging effects [105].

Arteriosclerosis is a common cardiovascular disease associated with chronic inflammation and ES dysfunction. Recent research has found that neutrophil microvesicles, by delivering miR-155 to ECs, drive the development of atherosclerosis [106]. Experimental results demonstrated that miR-155 in exosomes downregulated the expression of *SoS1*, a gene associated with fibroblast proliferation, and activated inflammatory pathways, thereby regulating the fibrotic response during cardiac injury [107]. Up-regulation of lncRNA 39868 expression in neutrophil-derived exosomes promoted the expression of p-AKT and Bcl-xL by acting on PDGFD and reduced the production of NADPH oxidase 2 (NOX2) and reactive oxygen species (ROS), alleviating ischemia-reperfusion-induced myocardial oxidative stress injury and improving heart function [108].

## 5. Exosome Therapy

The functional delivery of RNA molecules is an emerging vaccination and therapeutic approach, and mRNA delivery holds great clinical potential. Although naked mRNA delivery has been reported, its efficacy is limited by high plasma RNA degradation rates, low cellular uptake, and nucleic-acid-induced inflammatory responses. Formulating RNA into lipid nanoparticles (LNPs) has been developed as a method to overcome these limitations but LNPs and other synthetic nanoparticles are exogenous entities associated with a range of side effects. Exosomes are the only biologically normal nanovesicles, and compared to LNPs that can cause significant cellular toxicity, exosomes have shown no adverse effects in vitro or in vivo at any tested dose. However, the targeting ability of natural exosomes is limited, which partially restricts their application in cardiovascular disease treatment [109]. Constructing exosomes as drug delivery vehicles in a specific way can improve cardiac targeting and improve the stability of exosomes in the circulation [110]. Methods for engineering exosomes mainly include passive loading, active loading, and biotechnological modifications of donor cells. Each method has its own advantages and disadvantages, and the appropriate method should be selected based on the source, characteristics, and practical application of the exosome to effectively target delivery and stabilize its therapeutic effects. Passive loading involves co-incubation of the target drug with exosomes. Loading efficiency mainly depends on the concentration gradient and hydrophobicity of the drug. Compared to hydrophilic drugs, highly concentrated hydrophobic drugs have higher loading efficiency [111]. The advantage of passive loading is that it does not disrupt the integrity of the exosome membrane but the disadvantage is that the drug payload is lower compared to other loading methods.

Active loading involves directly targeting exosomes using techniques such as electroporation, ultrasound, liposomal freeze-thaw, and mechanical extrusion, etc. These methods are conducive to delivering various types of RNA to target tissues and regulate gene expression in target cells [112]. Some researchers have collected exosomes from cardiac progenitor cells and loaded them with miR-322 using electroporation [113]. Injection of these loaded exosomes into a myocardial infarction mouse model resulted in reduced infarct size, reduced fibrosis, and increased angiogenesis compared to mice injected with miR-322-unloaded exosomes. M2 macrophage-derived exosomes (M2-Exos) exhibited inflammation-targeting ability, and their active loading via electroporation significantly alleviated atherosclerosis [114]. Since exosomes are secreted by donor cells, biotechnological modification of donor cells can be utilized to change their genetic characteristics and enable the integration of molecular components naturally into budding vesicles, and substances internalized into the cell interior can be packaged into secreted exosomes. It has been reported that exosomes derived from MSCs can effectively promote cardiac injury repair. To further enhance the therapeutic effects of these exosomes, Wang and Mentkowski engineered donor cells via lentiviral transduction to produce ischemic myocardium-targeting peptide-lamp2b and cardiomyocyte-specific peptide-lamp2b fusion-expressing exosomes [109,115]; however, the targeting peptide-lamp2b fusion protein related to exosomes was found to be prone to degradation by nuclear proteases during exosome biogenesis. To overcome peptide loss, a glycosylation motif at different positions of the targeting peptide-lamp2b fusion protein was inserted to protect the peptide from degradation and increase the expression of the lamp2b fusion protein in cells and exosomes [116].

The advantages of exosomes over other carriers include biocompatibility and a lower likelihood of triggering innate and adaptive immune responses [117]. Clinical studies have demonstrated that exosomes secreted by dendritic cells can stimulate the immune system and, therefore, can be used as antitumor vaccines [118,119]. Exosome-encapsulated adeno-associated viral vectors are more efficient and less immunogenic than free vectors in delivering gene-therapeutic substances to recipient cells [120]. Manipulation of exosomes in vitro to load specific cargo (such as siRNAs, mRNAs, proteins, and miRNAs) and then using them as drugs or for bioengineering purposes have been reported [121,122]. Exosomes are naturally occurring nanocarriers that can maintain the biological activity of their cargo in living organisms. They possess low immunogenicity and high safety, making them promising for drug delivery. In addition, exosomes can circulate to all compartments in the body and have good potential for non-hepatic targeting of nucleic acid drug delivery. Currently, although preliminary results have been taken, there are several obstacles that need to be overcome for exosomes to become effective nucleic acid carriers, such as improving circulation time and in vivo distribution, enhancing organ-specific targeting, addressing safety concerns related to exosomes derived from tumors, and achieving stable large-scale production. The development of the exosomes industry is still at a very early and budding stage, offering tremendous development opportunities but also facing difficulties in clinical translation and inconsistent evaluation systems.

## 6. Conclusions

**Figure 2:**
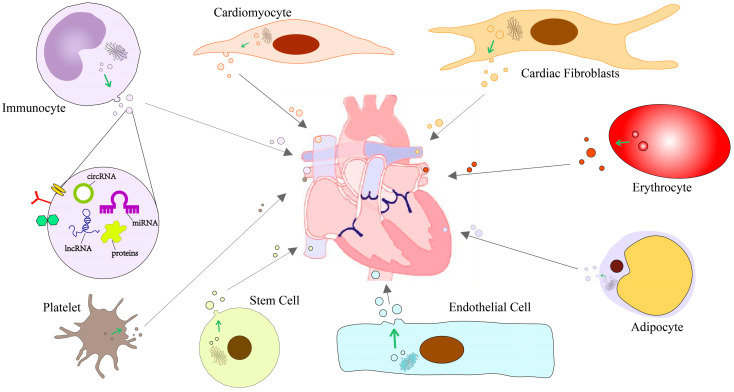
Schematic diagram of intercellular communication mediated by exosomal ncRNAs in cardiovascular disease.

Exosomes are an emerging area of research in cardiovascular disease. Currently, the study of exosomes highly relies on the methods used for isolation and purification, which impose higher requirements on the extraction steps and laboratory equipment. Several ncRNAs have been described in the context of cardiovascular disease specifically for mediating cellular communication (Table 1). The different small molecules carried by exosomes from different sources hold the potential to become therapeutic targets for future cardiovascular diseases and provide a basis for precision medicine (Figure 2). However, further research and exploration are still needed in this regard.

## Figures and Tables

**Table 1:** ncRNAs and Cardiovascular Diseases.

Exosomal ncRNA	Target	Cell Type/Communication	Refs.
miR-16	VEGF	Vascular smooth muscle cells–Endothelial cells	[53]
miR-155	miR-155	Vascular smooth muscle cells–Endothelial cells	[54]
circHIPK3	miR-106a-5p	Mouse aortic endothelial cells–Vascular smooth muscle cells	[55]
miR-92a-3p	THBS1	Endothelial cells–recipient Endothelial cells	[56]
miR-505	SIRT3	Vascular endothelial cells–Macrophages	[57]
miR-146a	Erbb4, Notch1, Irak1	Endothelial cells–Cardiomyocytes	[58]
miRNA-143/145	Unknown	Endothelial cells–Smooth muscle cells	[59]
LINC00174	SRSF1	Vascular endothelial cells–Myocardial cells	[60]
circNPHP4	miR-1231-EGFR	Monocytes–Coronary artery endothelial cells	[61]
miR-93	MMP12	Macrophages–Bronchial epithelial cells	[62]
miR-21_3p	SORBS2, PDLIM5	Cardiac fibroblasts–Cardiomyocytes	[69]
miR-23a-3p	SLC7A11	Cardiac fibroblasts–Cardiomyocytes	[70]
miR-200a-3q	VEGF-A/PIGF	Fibroblasts–Cardiac endothelial cells	[71]
miR-423-3p	RAP2C	Cardiac fibroblasts–Cardiomyocytes	[72]
circRNAbe3a	miR-138-5p/RhoC	M2 macrophages–Cardiac fibroblasts	[73]
lncRNA AK139128	Unknown	Cardiomyocytes–Fibroblasts	[74]
miR-208a	Dyrk2	Cardiomyocytes–Fibroblasts	[75]
miR-210-3p	GPD1L-PI3K/AKT	Myocytes–Fibroblasts	[76]
miR-92a	smad7	Cardiomyocytes–Myofibroblasts	[77]
miR-320	IGF-1, Hsp20, Ets2	Cardiomyocytes–Endothelial cells	[78]
circHIPK3	miR-29a-IGF-1	Cardiomyocytes–Cardiac microvascular endothelial cells	[80,81]
lncRNA-AK139128	Unknown	Cardiomyocytes–Cardiac fibroblasts	[74]
lncRNA-MALAT1	miR-92a-3p/ATG4a	Stem cells–Cardiomyocytes	[82]
miR-294	Unknown	Embryonic stem cells–Cardiomyocytes	[84]
miR-199a-3p	Akt-Sp1/p53 signaling pathway	Mesenchymal stem cells–Cardiomyocytes	[85]
miRNA-153-3p	VEGF/VEGFR2/PI3K/Akt/eNOS pathway	Mesenchymal stem cells–Cardiomyocytes	[86]
miRNA-21, miRNA-210	Unknown	Induced pluripotent stem cells–Cardiomyocytes	[87]
lncRNA-UCA1	miR-873-5p/XIAP	Mesenchymal stem cells–Cardiomyocytes	[88]
lncRNA KLF3-AS1	miR-138-5p/SIRT1	Mesenchymal stem cells–Cardiomyocytes	[89]
lncRNA H19	miR-675/VEGF, ICAM-1	Mesenchymal stem cells–Cardiomyocytes	[90]
lncRNA-NEAT1	miR-142-3p/FOXO1	Adipose mesenchymal stem cells–Macrophages	[91]
lncRNA-UCA1	miR-143/Bcl-2	Human umbilical cord mesenchymal stem cells–Cardiomyocytes	[92]
circRNA-0001273	Unknown	Umbilical cord mesenchymal stem cells–Myocardial cells	[93]
circRNA_0002113	miR-188-3p/RUNX1 axis	Mesenchymal stem cells–Myocardial cells	[94]
circHIPK3	miR-421/FOXO3a	Umbilical cord mesenchymal stem cells	[95]
miR-27b-3p	PPARα	Visceral adipocytes–Endothelial cells	[99]
miR-23a-3p	RAP1	Epididymal white adipose tissue–Myocardial fibrosis	[100]
lncRNA-SNHG9	TRADD	Adipocytes–Endothelial cells	[101]
miRNA-31	FIH1	Adipose-derived stem cells–Cardiomyocytes	[102]
miR-802-5p	HSP60	Adipocytes–Cardiac myocytes	[103]
miR-142-3p	WNT signaling pathway	CD4+ T cells–Cardiac myofibroblasts	[104]
miR-34a-5p	PNUTS signaling pathway	Cardiomyocytes–Macrophages	[105]
miR-155	VEGF signaling pathway	Neutrophil microvesicles–Endothelial cells	[106]
miR-155	Son of Sevenless 1	Macrophages–Fibroblasts	[107]
lncRNA 39868	PDGFD	Neutrophils	[108]

## Data Availability

Not applicable.
